# Determination of Nursing Students' Opinions on the Use of Animal‐Assisted Interventions in Pediatric Care and Their Animal Love Levels

**DOI:** 10.1111/nhs.70128

**Published:** 2025-05-14

**Authors:** Abdullah Sarman, Veysel Can, Suat Tuncay, Ahmed Loutfy

**Affiliations:** ^1^ Department of Pediatric Nursing, Faculty of Health Science Bingöl University Bingöl Turkey; ^2^ Faculty of Health Science Van Yüzüncü Yıl University Van Turkey; ^3^ Department of Nursing, College of Health Sciences University of Fujairah Fujairah UAE; ^4^ Department of Pediatric Nursing, Faculty of Nursing Beni‐Suef University Beni Suef Egypt

**Keywords:** animal love, animal‐assisted intervention, child, nursing students

## Abstract

Animal‐assisted intervention (AAI) integrates animals into health, education, and human services for therapeutic benefits. This study examined nursing students' perspectives and affinity for animals regarding AAI in pediatric care. A descriptive cross‐sectional design was used, involving nursing students from a university's Health Sciences Faculty. Data were collected via a sociodemographic form and the Öney Animal Love Scale and analyzed using the R programming language. Results showed that 62.6% of participants were female, 30% were first‐year students, and 18.7% owned pets. While 83.7% lacked prior AAI knowledge, 4.2% had used AAI. Despite limited awareness, 72.7% found AAI effective, and 52.2% considered it beneficial for children. A mediation analysis indicated that pet ownership positively influenced students' affinity for animals and belief in AAI's effectiveness. Prior AAI experience strengthened this relationship. In conclusion, nursing students had limited AAI knowledge but recognized its benefits. Pet ownership and prior exposure to AAI were key factors in fostering positive attitudes toward its role in pediatric care.

**Summary**

- Most nursing students recognize the potential effectiveness of animal‐assisted interventions (72.7%) and support their use in pediatric care (52.2%).
- Limited knowledge of AAI is evident among students, with 83.7% reporting no prior awareness of the method.
- Pet ownership and previous AAI experience positively influence students' attitudes and belief in the intervention's effectiveness.

## 1 Introduction

Animal‐assisted intervention (AAI) is a structured intervention with specific goals that intentionally involves animals in health, education, and human services to achieve therapeutic benefits in humans (Howe et al. 2020). In recent years, the use of AAI in the health field has been gradually increasing (Sarman and Günay 2023; Sarman and Tuncay 2024). This growing interest is, particularly, evident in pediatric care, where children often experience high levels of distress and anxiety during medical procedures. Studies have shown that AAI can significantly reduce procedural anxiety and pain perception in pediatric patients, making it a valuable complementary approach in clinical settings (Calcaterra et al. 2015; Hinic et al. 2019). One significant reason for this trend is the potential of AAIs to support children's physical (Wolan‐Nieroda et al. 2020), emotional (López‐Fernández et al. 2024), and social development (Wijker et al. 2020). Studies have shown that AAIs can have positive effects on emotional well‐being (Jalongo and Guth 2022; Sarman and Günay 2023), motivation (Wohlfarth et al. 2013), and communication skills (Lavín‐Pérez et al. 2023) especially in children. For instance, therapy animals have been found to decrease fear and distress in hospitalized children, leading to improved cooperation during medical examinations and treatments (Urbanski and Lazenby 2012). Research by Calcaterra et al. (2015) concluded that AAI is an effective method for reducing children's stress and pain (Calcaterra et al. 2015). In a study conducted by Hinic et al. (2019), it was reported that anxiety significantly decreased in both children and their parents who practiced this method (Hinic et al. 2019). Although the precise mechanisms by which animals produce this effect remain unclear, theories such as “biophilia,” “attachment,” “verbal‐symbolic system,” “motivation,” and “social support” have been proposed to explain the underlying mechanisms (Beetz 2017). The biophilia hypothesis suggests that humans have an innate tendency to connect with nature and living beings, which can have a calming effect and reduce stress in pediatric patients (Fine and Weaver 2018). In hospital settings, interactions with therapy animals may help children feel more at ease by fulfilling this intrinsic need for connection with living organisms. Similarly, the attachment theory posits that humans form emotional bonds with animals in ways similar to human relationships, providing children with a sense of security and comfort during medical procedures (Bowlby 1982). This is, particularly, relevant in pediatric care, where children may experience fear and distress in unfamiliar medical environments. The presence of an animal can serve as a secure base, helping them cope with medical stressors and promoting emotional resilience. The verbal‐symbolic system theory highlights how animals can serve as a medium for communication, especially for children who struggle to express their emotions verbally (Kruger and Serpell 2010). In pediatric care, this mechanism allows children to externalize their fears and anxieties through interactions with animals, which can facilitate better communication with healthcare providers. Moreover, the motivation theory suggests that the presence of an animal can encourage children to participate in therapeutic activities or medical treatments they might otherwise resist. For example, therapy animals have been shown to increase engagement in physical rehabilitation exercises and social interactions, enhancing overall treatment outcomes (Beetz 2017). Finally, the social support theory suggests that animals act as nonjudgmental companions, reducing loneliness and providing emotional support to pediatric patients (Fine and Weaver 2018). By offering unconditional acceptance, animals can help children navigate the emotional challenges associated with illness and hospitalization. Upon examining these theories, it becomes evident that the concept of animals normalizing the hospital experience for patients and their families, empowering children to actively engage in their treatment, and promoting happiness by alleviating pain, anxiety, and fear is embraced (Wu et al. 2002).

It is crucial for nursing students, especially those bound for healthcare careers, to be knowledgeable about effective nondrug methods for pediatric care (Al‐Yateem et al. 2016). These methods, proven to be effective, should be seen as complementary therapies and incorporated into patient care whenever possible (Sarman and Günay 2023). To promote the adoption of such practices, it is essential to explore the perspectives of nurses and nursing students, who represent the future of healthcare, as well as their affinity toward animals (Acquadro Maran et al. 2022). Understanding nursing students' opinions in this area and their fondness for animals could aid in developing future educational programs. Despite numerous studies in the literature (Correale et al. 2022; Sarman and Tuncay 2024; Tedeschi et al. 2015), none have yet delved into nursing students' attitudes toward using AAIs in pediatrics or their affection for animals.

The purpose of this study was to investigate nursing students' perspectives regarding the utilization of AAIs in pediatric care and their affinity toward animals. To better understand the relationship between nursing students' affinity for animals and their perspectives on AAI, mediation analysis was incorporated into this study. The mediation model aimed to assess whether the level of animal love, as measured by the Öney Animal Love Scale, mediates the relationship between demographic characteristics (e.g., pet ownership, gender) and students' attitudes toward AAI in pediatric care. Previous research has suggested that individuals with a greater affinity for animals are more likely to support and engage with AAI (Fine et al. 2019). Therefore, this study hypothesized that the degree of animal love may act as a mediating factor, influencing students' willingness to implement AAI in clinical practice. The findings from this study are anticipated to provide insights into the knowledge level and educational requirements of nursing students in this domain, thus aiding in the enhancement of future educational initiatives.

## 2 Methods

### 2.1 Study Design

This study was a descriptive cross‐sectional study.

### 2.2 Place and Time

This study was carried out with students from the Nursing Department at the Faculty of Health Sciences of a university situated in eastern Turkey. The fieldwork took place between January 25, 2024, and February 25, 2024.

### 2.3 Population and Sample

The study's population consisted of all students from the Department of Nursing at Bingöl University Faculty of Health Sciences. A convenience sampling method was used, as participation was voluntary and limited to students who agreed to take part in the study. To maximize participation, course schedules were examined to identify high‐intensity class days, and information about the study was shared with a total of 400 students on Mondays, Wednesdays, and Fridays when class density was high. Among these, 337 students voluntarily participated in the study, resulting in a response rate of 84.25%. The remaining 63 students did not participate, either due to lack of interest, time constraints, or personal reasons, but specific dropout reasons could not be formally documented. Following the research's conclusion, a post hoc power analysis was conducted utilizing the G*Power 3.1 package program and OpenEpi Version 3 program. Literature suggests that for descriptive and cross‐sectional studies, determining the sample size can be based on an effect size of 0.5, an alpha level (*α*) of 0.05, and a power range of 0.80 (1 – *β*) (Cohen 1992). In the power analysis involving 337 participants, a confidence interval of 95%, an alpha of 5%, and a power of 80% were utilized.

### 2.4 Inclusion Criteria

All nursing students over 18 years of age, able to communicate, and capable of filling out an online questionnaire were included in the study.

### 2.5 Exclusion Criteria

The data of nursing students who filled out the questionnaire inconsistently and incompletely were excluded.

### 2.6 Data Collection

The research data were gathered utilizing the “Socio‐Demographic Characteristics Form” and the “Öney Animal Love Scale.” To broaden the reach of data collection, questionnaire forms were designed using Google Forms for online dissemination. The form's link was distributed to student nurses through various social media platforms such as WhatsApp, Telegram, text messages, and emails. Participants were requested to complete the data collection instruments after consenting to the voluntary participation form provided through the online survey.

### 2.7 Data Collection Tools

#### 2.7.1 Sociodemographic Characteristics Form

This form was developed by the researchers based on the literature (Sarman and Günay 2023; Sarman and Tuncay 2024) and encompasses inquiries regarding age, gender, marital status, socioeconomic class, educational attainment, willingness to engage with the department, income level, household composition, pet ownership status, familiarity with AAI, prior participation in AAI activities, perspectives on the efficacy of AAI, and opinions on the suitability of different animals for clinical application within the framework of AAI.

#### 2.7.2 Öney Animal Love Scale

The “Öney Animal Love” Scale, developed by Önal et al. (2020), comprises 28 items and is structured into five subdimensions (Önal et al. 2020). These dimensions include aspects such as “positive interaction,” “social rights,” “sensitivity,” “discrimination,” and “medical purpose” concerning animals. Scale items are scored between 1 and 5. The number of positive items is 21 and the number of negative items is 7. The scores obtained from the positive interaction (13, 18, 20, 21, 22, 24, 25, 26, 27), social rights (2, 3, 5, 6, 7, 10), and sensitivity (1, 4, 8, 9, 11, 12) subdimensions consisting of positive statements should be summed directly, while the discrimination (14, 15, 19, 23, 28) and medical purpose (16, 17) subdimensions consisting of negative statements are inverted and summed. The cut‐off point of the scale was determined by considering the 25th and 75th percentile values that are frequently used in practice. Scores on the scale range from 0 to 94, indicating a “low level of animal love”; 95–116, indicating a “moderate level of animal love”; and 117–140, indicating a “high level of animal love.” In the validity and reliability study conducted by Önal et al. (2020), the Cronbach's alpha value of the scale was found to be 0.89 (Önal et al. 2020). In this study, the Cronbach's alpha internal consistency coefficient was found to be 0.886.

### 2.8 Data Analysis

The data obtained in the study were evaluated using the IBM SPSS Statistics 26.0 program. A database was created, and error analysis was conducted. Descriptive statistics were employed for data analysis. Normality of the variables was assessed using Skewness and Kurtosis tests. For variables meeting the conditions of normal distribution, analyses including independent groups *t* tests and analysis of variance (ANOVA) were performed. Nonnormal distributions were addressed using nonparametric tests such as the Mann–Whitney *U* test, Kruskal–Wallis *H* analysis, and Spearman tests. Mediation analysis was conducted to examine whether the affinity for animals (measured using the Öney Animal Love Scale) mediates the relationship between nursing students' demographic characteristics and their attitudes toward AAI. This analysis was chosen because previous studies have indicated that individuals' personal experiences with animals influence their perception of AAI, potentially acting as a mediator in shaping attitudes. By including this analysis, the study aimed to identify whether animal affinity strengthens or weakens the correlation between demographic variables (e.g., pet ownership, gender) and attitudes toward AAI. Mediation analysis was carried out using the R programming language version 4.1.3, utilizing the mediation analysis method developed by Andrew Hayes (Hayes 2009). Statistical significance was determined at *p* < 0.05 for all analyses.

## 3 Results

It was determined that 62.6% of the students were female, 30% were in the first grade, and 95.8% were high school graduates. Additionally, 78% of the students had a medium income, and 75.4% had a nuclear family structure. The rate of preference for the department was 56.7% (Table 1).

**TABLE 1:** Distribution of sociodemographic characteristics of the students according to their mean scale scores.

Variable	*n*	%	PI *X̄* ± SD	SR *X̄* ± SD	*S X̄* ± SD	*D X̄* ± SD	MP *X̄* ±S D	Total *X̄* ± SD
Gender								
Male	126	37.4	16.7 ± 6.9	16.6 ± 5.5	10.5 ± 4.1	16.9 ± 3.8	6.9 ± 2.3	70.8 ± 13.4
Female	211	62.6	19.2 ± 8.7	17.3 ± 5.8	11.8 ± 5.5	18.7 ± 3.8	8.1 ± 2.2	73.2 ± 18.4
*t* (*p*)			2.9186 (0.003)	0.858 (0.286)	4.850 (0.019)	1.424 (< 0.001)	0.911 (< 0.001)	5.386 (0.048)
Cohen's *d*			−0.310	−0.120	−0.260	−0.47	−0.540	−0.140
Grade								
1	101	30.0	17.5 ± 8.6	15.8 ± 6.3	11.2 ± 5.5	17.6 ± 3.4	7.2 ± 2.4	68.9 ± 17.3
2	74	22.0	16.2 ± 7.0	15.7 ± 5.9	9.8 ± 4.8	17.8 ± 4.1	7.4 ± 2.3	69.7 ± 17.6
3	80	23.7	17.5 ± 7.6	18.1 ± 4.7	11.4 ± 3.6	17.9 ± 3.4	7.6 ± 2.1	73.1 ± 13.3
4	82	24.3	19.2 ± 7.1	18.2 ± 4.7	11.4 ± 4.4	18.9 ± 4.5	8.1 ± 2.3	74.1 ± 12.1
*F* (*p*)			1.955 (0.121)	4.981 (0.002)	2.085 (0.102)	1.763 (0.154)	3.273 (0.021)	2.117 (0.098)
Bonferroni test				3 > 1 = 2 = 4			2 = 4 > 3 = 2	
*η* ^2^			0.0173	0.0429	0.0184	0.0156	0.0286	0.0187
Education level								
Undergraduate education in progress^a^	323	95.8	16.1 ± 6.7	13.1 ± 5.1	9.5 ± 3.7	17.8 ± 5.1	6.8 ± 2.5	64.1 ± 14.4
Previously finished a bachelor's degree^b^	12	3.6	17.7 ± 7.6	13.7 ± 3.6	11.1 ± 4.7	18.1 ± 3.8	7.5 ± 2.6	71.8 ± 15.3
Postgraduate^c^	2	0.6	19.1 ± 12.2	17.2 ± 5.6	12.8 ± 6.6	19.7 ± 3.0	7.7 ± 2.3	72.4 ± 20.6
Kw*X* ^*2*^ (*p*)			0.787 (0.675)	12.879 (0.002)	2.478 (0.290)	1.889 (0.389)	1.191 (0.551)	4.836 (0.089)
Games–Howell test				*a* > *b* = *c*				
*η* ^2^			−0.0036	0.0326	0.0014	−0.0003	−0.0024	0.0085
Marital status								
Married	4	1.2	19.1 ± 4.5	16.9 ± 5.6	11.1 ± 4.7	18.1 ± 3.8	7.7 ± 2.3	71.5 ± 15.4
Single	333	98.8	17.6 ± 7.7	15.1 ± 8.1	8.7 ± 2.5	13.1 ± 5.1	4.2 ± 2.1	60.1 ± 1.9
*Z* (*p*)			−1.144 (0.253)	−0.866 (0.387)	−0.476 (0.634)	−1.038 (0.299)	−1.950 (0.051)	−2.568 (0.010)
Cohen's *d*			0.200	0.220	0.950	0.980	1.670	4.770
Income status								
Low^a^	60	17.8	17.1 ± 8.3	17.1 ± 7.1	11.1 ± 5.4	17.1 ± 4.6	7.4 ± 2.1	69.8 ± 21.1
Middle^b^	263	78.0	17.5 ± 7.3	16.8 ± 5.2	10.8 ± 4.5	18.1 ± 3.3	7.4 ± 2.3	71.2 ± 13.7
High^c^	14	4.2	23.2 ± 10.1	18.6 ± 6.3	13.2 ± 3.8	18.2 ± 3.7	7.7 ± 2.3	80.5 ± 16.4
Kw*X* ^*2*^ (*p*)			7.001 (0.030)	2.268 (0.322)	7.133 (0.028)	2.460 (0.292)	1.164 (0.559)	7.330 (0.026)
Games–Howell test			*c* > *b* = *a*		*c* > *a* > *b*			*c* > *b* > *a*
*η* ^2^			0.0402	0.0134	0.041	0.0145	0.0069	0.042
Family type								
Extended family^a^	78	23.1	19.1 ± 14.9	16.9 ± 5.4	12.8 ± 9.9	20.1 ± 4.8	7.9 ± 2.3	74.8 ± 37.3
Nuclear family^b^	254	75.4	18.8 ± 8.8	16.8 ± 6.1	11.5 ± 4.7	18.3 ± 3.7	7.1 ± 2.4	71.3 ± 13.5
Broken family^c^	5	1.5	17.2 ± 7.2	16.1 ± 10.1	10.8 ± 4.6	17.1 ± 4.4	6.9 ± 2.3	71.2 ± 19.1
Kw*X* ^*2*^ (*p*)			1.212 (0.546)	0.180 (0.914)	1.846 (0.397)	5.455 (0.065)	13.753 (0.001)	1.392 (0.499)
Games–Howell test							*a* > *c* > *b*	
*η* ^2^			0.0072	0.0011	0.0109	0.0316	0.0761	0.0083
Preferring the department willingly								
Yes	191	56.7	18.1 ± 8.1	17.4 ± 5.7	11.1 ± 4.8	18.4 ± 3.7	7.7 ± 2.2	71.8 ± 15.9
No	146	43.3	17.2 ± 7.4	16.5 ± 5.5	10.9 ± 4.6	17.5 ± 4.1	7.5 ± 2.5	71.1 ± 15.1
*t* (*p*)			1.062 (0.289)	1.436 (0.152)	0.435 (0.664)	2.268 (0.024)	0.813 (0.417)	0.491 (0.624)
Cohen's *d*			0.120	0.160	0.040	0.230	0.090	0.040
	*X̄* ± SD							
Age	21.25 ± 2.60							

*Note:* Undergraduate education in progress is abbreviated as ^a^. Previously finished a bachelor's degree is abbreviated as ^b^. Postgraduate is abbreviated as ^c^.

Abbreviations: *D*, discrimination; *F*, ANOVA test; Kw*X* ^*2*^, Kruskal–Wallis *H* test; MP, medical purpose; PI, positive interaction; *S*, sensitivity; SR, social rights; *t*, Student‘s *t* test; *Z*, Mann–Whitney *U* test.

Further analysis revealed that the mean scores of the discrimination and medical purpose subscales, as well as total scale scores, were higher among women. Similarly, the mean score of the medical purpose subdimension was found to be higher among senior students. Married individuals exhibited a higher mean total score for animal love. Moreover, the mean scores of positive interactions, sensitivity subdimension, and total scores were higher among those who reported a high‐income status. It was determined that students living in extended families had higher scores in the medical purpose subdimension. Additionally, the discrimination subdimension mean scores were higher among students who willingly preferred their department (Table 1).

It was determined that 18.7% of the nursing students had pets, 83.7% had no prior knowledge about AAI, 4.2% had previously utilized AAI methods, 72.7% believed AAI to be effective, and 52.2% expressed belief that this method could be beneficial for children. Additionally, 29.5% of the students stated that animals could aid in general adaptation to the hospital environment. Among the animals considered for AAI, aquarium fish were the most commonly suggested, with 23.3% of respondents in agreement (Table 2).

**TABLE 2:** Distribution of students' thoughts on AAI according to scale mean scores.

Variable	*n*	%	PI *X̄* ± SD	SR *X̄* ± SD	*S X̄* ± SD	*D X̄* ± SD	MP *X̄* ± SD	Total *X̄* ± SD
Feeding pets								
Yes	63	18.7	17.8 ± 7.5	17.6 ± 5.5	11.2 ± 4.4	19.1 ± 4.1	7.6 ± 2.3	72.2 ± 15.3
No	274	81.3	16.9 ± 8.5	13.9 ± 5.3	10.3 ± 5.6	17.8 ± 3.8	7.5 ± 2.5	67.8 ± 15.8
*t* (*p*)			−0.862 (0.389)	−4.865 (< 0.001)	−1.333 (0.183)	2.223 (0.027)	−0.022 (0.983)	2.012 (0.045)
Cohen's *d*			0.108	0.693	0.167	0.337	0.041	0.280
Having knowledge about AAI								
Yes	55	16.3	17.9 ± 7.2	17.2 ± 5.5	11.1 ± 4.4	19.2 ± 4.6	7.6 ± 2.3	71.8 ± 14.1
No	282	83.7	16.3 ± 9.7	15.4 ± 6.2	10.4 ± 5.8	17.8 ± 3.7	7.6 ± 2.5	69.1 ± 21.3
*t* (*p*)			−1.419 (0.157)	−2.176 (0.030)	−0.992 (0.322)	2.331 (0.020)	−0.178 (0.859)	−1.245(0.214)
Cohen's *d*			0.171	0.295	0.125	0.363	0.000	0.133
Previously using the AAI method								
Yes	14	4.2	17.7 ± 12.5	17.1 ± 5.5	12.2 ± 7.4	19.9 ± 4.7	7.9 ± 2.6	72.1 ± 26.1
No	323	95.8	17.6 ± 7.5	14.2 ± 6.5	10.9 ± 4.6	17.9 ± 3.8	7.6 ± 2.3	71.3 ± 14.9
*Z* (*p*)			−1.329 (0.184)	−1.959 (0.050)	−0.055 (0.956)	−1.692 (0.091)	−0.477 (0.633)	−1.564 (0.118)
Cohen's *d*			0.013	0.449	0.274	0.521	0.130	0.052
Do you think the AAI is effective?								
Yes	245	72.7	20.3 ± 7.6	18.1 ± 5.7	12.3 ± 5.3	18.6 ± 3.5	8.1 ± 2.2	74.4 ± 18.6
No	92	27.3	16.6 ± 7.5	16.5 ± 5.5	10.5 ± 4.4	16.4 ± 4.4	7.3 ± 2.5	70.2 ± 13.9
*t* (*p*)			−3.924 (< 0.001)	−2.149 (0.032)	−3.178 (0.002)	4.798 (< 0.001)	1.780 (0.046)	−2.229 (0.026)
Do you think AAI can be used in children?								
Yes	176	52.2	19.3 ± 8.6	17.3 ± 5.9	11.5 ± 5.1	18.5 ± 3.6	7.6 ± 2.3	73.4 ± 17.4
No	161	47.8	16.1 ± 6.5	16.5 ± 5.3	10.5 ± 4.3	17.5 ± 4.2	7.6 ± 2.3	69.5 ± 13.2
*t* (*p*)			−3.948 (< 0.001)	−1.208 (0.228)	−1.804 (0.072)	2.508 (0.013)	0.039 (0.969)	−2.306 (0.022)
Cohen's *d*			0.417	0.142	0.211	0.257	< 0.001	0.251
What are the issues that you think AAI has an impact on children?								
Pain management^a^	42	23.9	15.6 ± 6.7	15.9 ± 5.1	10.4 ± 4.1	18.4 ± 3.7	7.5 ± 3.7	68.1 ± 15.3
Fear management^b^	46	26.1	17.1 ± 6.3	17.1 ± 5.4	10.7 ± 4.3	18.3 ± 3.6	8.1 ± 2.3	71.4 ± 11.6
Anxiety management^c^	36	20.5	16.4 ± 7.1	17.9 ± 5.2	11.1 ± 5.3	18.4 ± 3.6	7.2 ± 2.3	71.1 ± 14.2
Ensuring general adaptation to the hospital^d^	52	29.5	15.3 ± 6.1	15.6 ± 5.4	10.1 ± 4.1	19.1 ± 3.4	7.7 ± 2.3	68.1 ± 12.1
*F* (*p*)			4.234 (0.002)	1.445 (0.219)	1.052 (0.380)	1.858 (0.118)	0.661 (0.619)	1.819 (0.125)
Bonferroni test			*b* > *c* > *a* = *d*					
*η* ^2^			0.069	0.025	0.018	0.031	0.011	0.031
Which animals are eligible for AAI for children?								
Cats^a^	27	15.3	16.8 ± 7.9	15.6 ± 5.6	10.0 ± 3.9	17.4 ± 4.1	6.1 ± 2.3	66.1 ± 12.3
Dogs^b^	26	14.8	16.8 ± 7.2	18.0 ± 5.2	10.4 ± 4.8	18.9 ± 4.1	8.3 ± 2.2	71.7 ± 10.4
Aquarium fishes^c^	41	23.3	16.4 ± 5.9	17.5 ± 5.1	11.5 ± 4.2	18.3 ± 3.1	8.4 ± 2.3	72.5 ± 15.7
Birds^d^	13	7.3	17.1 ± 8.8	16.3 ± 6.1	11.8 ± 6.1	16.7 ± 2.9	6.4 ± 2.8	68.7 ± 21.7
Poultry^e^	36	20.5	14.6 ± 6.2	17.1 ± 4.9	10.0 ± 4.3	19.3 ± 3.1	7.7 ± 2.1	69.7 ± 13.8
Farm animals^f^	33	18.8	15.6 ± 4.9	14.5 ± 5.2	10.1 ± 5.1	17.5 ± 4.2	8.0 ± 2.1	67.6 ± 9.4
Kw*X* ^*2*^ (*p*)			4.234 (0.516)	10.052 (0.074)	5.793 (0.327)	10.860 (0.054)	20.926 (0.001)	12.016 (0.035)
Games–Howell test							*e* = *b* = *f*> *c* > *d* = *a*	*c* > *b* >*e* > *d* > *f* > *a*
*η* ^2^			0.111	0.228	0.146	0.242	0.381	0.261

*Note:* Pain management is abbreviated as ^a^. Fear management is abbreviated as ^b^. Anxiety management is abbreviated as ^c^. The expression Ensuring general adaptation to the hospital is abbreviated as ^d^.

Abbreviations: *D*, discrimination; *F*, ANOVA test; Kw*X* ^*2*^, Kruskal–Wallis *H* test; MP, medical purpose; PI, positive interaction; *S*, sensitivity; SR, social rights; *t*, Student's *t* test; *Z*, Mann–Whitney *U* test.

The mean scores of the social rights and discrimination subdimensions, as well as the total scores, were found to be higher among students who owned pets. Similarly, those who were knowledgeable about AAI exhibited higher scores in the social rights and discrimination subdimensions. Students who perceived AAI as effective scored higher in the scale subdimension and total scores. Additionally, those who believed AAI could be utilized for children showed higher scores in positive interaction and discrimination subdimensions. Moreover, students who endorsed animals as effective in anxiety management demonstrated higher scores in the positive interaction subdimension. Finally, students who advocated for the use of aquarium fish in AAI displayed higher scores in the medical purpose subdimension and total score (Table 2). It was determined that the mean score of the Öney Animal Love Scale for nursing students was 71 ± 15.47, indicating a low level of animal love.

**FIGURE 1:**
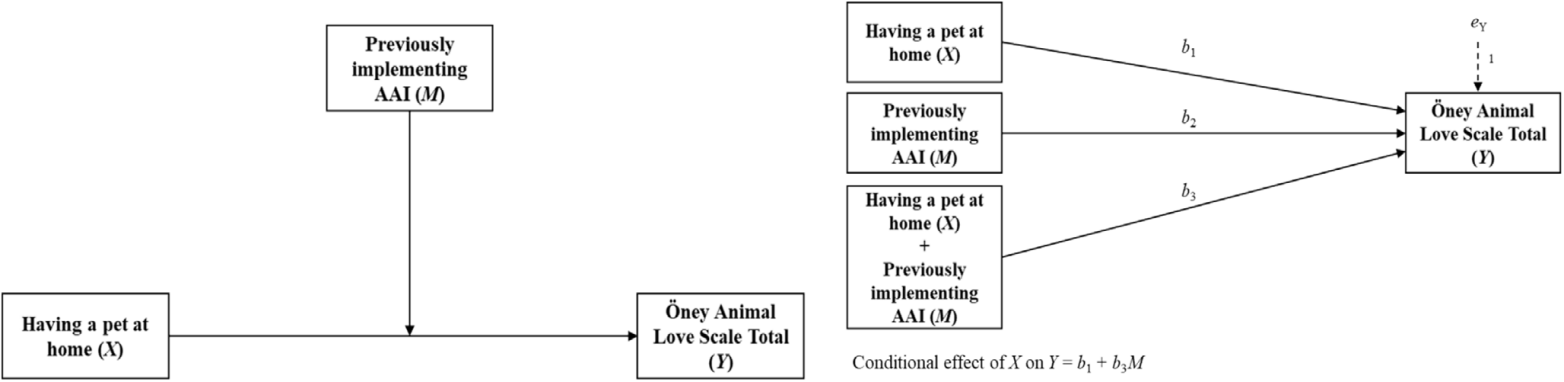
The mediating role of having a pet at home and having previously practiced AAI on the scale total score. Statistical model of simple mediation analysis (Model 1).

In this study, we investigated the mediating role of pet ownership and prior engagement in AAI on the overall score of the scale (Figure 1). To accomplish this, direct regression analysis was conducted between the variables, and the results are outlined in Table 3. The data revealed significant associations between pet ownership, AAI engagement, and the total scale score. Specifically, having a pet at home, prior engagement in AAI, and the combined effect of both were found to correlate with the total scale score (Table 3). These results were derived using the bootstrap method with 5000 replications, indicating a direct influence of pet ownership and prior AAI engagement on affection toward animals. To further clarify the mediation model, direct and indirect effects were analyzed through regression. The dependent variable was the Öney Animal Love Scale total score, while the independent variable was pet ownership (*X*), and the mediator was prior AAI experience (*M*). Potential covariates such as age, gender, and socioeconomic status were not controlled in this model; however, future studies could benefit from examining these factors. As shown in Table 3, regression analyses revealed that pet ownership (*X*) had a significant effect on the total scale score (*β* = 1.320, *p* = 0.032). Similarly, prior AAI experience was also found to have a significant effect on the scale score (*β* = 0.108, *p* = 0.011). When both factors were considered together (*X* + *M*), the effect increased to *β* = 1.428 (*p* = 0.043). These findings indicate that pet ownership and AAI experience significantly influence emotional attitudes toward animals. The bootstrap method with 5000 samples was used to estimate confidence intervals, ensuring the robustness of the model. The LLCI and ULCI values (Table 3) support the statistical significance of the regression coefficients. The *R* ^2^ values indicate that the independent variables explain 32.7% of the variance, suggesting a moderate explanatory power of the model. Figure 1 illustrates the mediation model, depicting the direct and indirect effects of pet ownership (X), prior AAI experience (M), and their combined effect on the total scale score. The analysis confirms that pet ownership and AAI experience have a direct impact on animal affection and, when combined, exert an even stronger effect.

**TABLE 3:** Mediation Analysis Model 1: The mediating role of having pets at home and having practiced AAI before on the scale total score.

Variable	*β*	SE	*t*	*p*	*R* ^2^	*F*	LLCI	ULCI
Having a pet at home (*X*)	1.320	2.184	2.147	0.032	0.457	1.767	0.393	8.987
Previously implementing AAI (*M*)	0.108	4.665	0.023	0.011	0.327	2.145	−9.068	9.284
(*X + M*)	1.428	0.844	4.473	0.043	0.443	1.063	69.659	72.981

Abbreviations: LLCI, lower limit of the confidence interval; SE, standard error; ULCI, upper limit of the confidence interval.

## 4 Discussion

In this study, aimed at assessing nursing students' perspectives on the use of AAIs in pediatric care and their affinity toward animals, it was found that students exhibited a low level of affection toward animals. This finding suggests that nursing students lack the desired positive outlook on employing AAIs in pediatric settings. Furthermore, gender analysis revealed that females had a significantly higher mean score, indicating a greater affinity toward animals compared to males. Herzog (2007) highlighted potential gender disparities in human–animal interaction, with women displaying more positive behaviors and attitudes toward animals, such as their use in therapy and animal welfare, while men often exhibit more negative attitudes and behaviors, including hunting and animal mistreatment (Herzog 2007).

One of the significant findings of this study was the correlation between pet ownership and higher total scores on the Öney Scale. This phenomenon is believed to stem from the emotional bond formed with an animal. This relationship may be attributed to several factors, including the emotional bonding process, increased exposure to caregiving, and the development of empathy through daily interactions with animals. Research suggests that individuals who own pets experience higher levels of emotional connection with animals due to consistent interaction, which fosters attachment and a sense of companionship (Zilcha‐Mano et al. 2011). Furthermore, pet ownership requires caregiving responsibilities such as feeding, grooming, and ensuring the pet's well‐being, which, in turn, strengthens an individual's perception of animals as sentient beings rather than mere objects (Barker and Wolen 2008). Exposure to caregiving behaviors has been shown to enhance empathy, social sensitivity, and a deeper appreciation for animal welfare (Prato‐Previde et al. 2022). Thus, students with prior experience in caring for pets may be more inclined to recognize the benefits of AAIs and demonstrate a higher level of affinity toward animals. In the literature concerning attachment to pets, it has been documented that such attachment is evident in both males and females, albeit with slight variations based on gender. Melson et al. (1991) asserted that attachment to pets encompasses behavioral, cognitive, and emotional dimensions, which subsequently influence the adoption of the animal (Melson et al. 1991). Additionally, Lass‐Hennemann et al. (2022) noted a direct association between emotional attachment to pets and mental well‐being, highlighting that attachment style influences the quality of the relationship with the animal (Lass‐Hennemann et al. 2022). It was determined that nursing students who expressed the belief that AAI was an effective method and could be beneficial for children exhibited a higher affinity for animals. Furthermore, a majority of the nursing students acknowledged the positive impact animals can have on children's management of fear, and they stated that such interventions could enhance children's treatment processes. In a study conducted by Lapian et al. (2023), it was reported that human–animal interaction positively influenced both patients and hospital staff. The study highlighted the positive social, emotional, and physiological effects of animals, suggesting that such methods could be utilized to create a conducive and comforting environment for both patients and healthcare team members (Lapian et al. 2023). Participants who advocated for the use of aquarium fish within the context of AAI demonstrated a higher level of affection for animals. This result could be attributed to the widespread presence of aquariums in various settings such as homes and offices, as well as the relative ease of maintenance associated with aquarium fish compared to other animals. However, the finding that only 16.3% of nursing students possessed knowledge about AAI points to the inadequacy in the effectiveness and usability of such nondrug methods in training. Nimer and Lundahl (2007) asserted that despite the longstanding application and known effectiveness of AAIs, participants fail to sufficiently utilize them, highlighting a need for more information on this subject (Nimer and Lundahl 2007). Moreover, cultural attitudes toward animals may have played a role in shaping the students' perspectives on AAIs. In particular, regional differences within Turkey, such as those observed in eastern Turkey, could influence individuals' views on human–animal interactions. Research has indicated that in some rural and conservative communities, animals are traditionally perceived as utilitarian beings rather than as companions (Serpell 2004). Additionally, religious and societal beliefs may contribute to the way people relate to animals, with some communities exhibiting hesitancy toward close human–animal interactions due to concerns about cleanliness and religious interpretations regarding animal handling (Pasaribu et al. 2021). These cultural influences may partially explain the lower levels of affinity toward animals observed among nursing students and their reluctance to embrace AAIs in pediatric care. These results underscore the importance of incorporating AAIs more prominently into nursing education programs and better preparing students in this area while also considering cultural influences that may shape their perceptions. Future studies could explore how cultural and regional factors impact attitudes toward AAIs and develop strategies to address potential barriers in different sociocultural contexts.

## 5 Limitations

This study is subject to certain limitations. First, it was conducted exclusively with nursing students from a single university, thereby constraining its generalizability to the broader population. Moreover, the utilization of a self‐report scale to gauge attitudes toward animal affection restricted the ability to ascertain the overall disposition of the students. However, despite these constraints, the study's strengths lie in its extensive coverage of the population, encompassing all classes, and facilitating interclass comparisons. Furthermore, the study's novelty in examining student nurses' perspectives on the utilization of AAIs in children, alongside their levels of affection toward animals, contributes significantly to the existing body of literature.

## 6 Relevance for Clinical Practice

- Given the observed gap in knowledge about AAIs among nursing students, there is a critical need to integrate comprehensive AAI‐related content into nursing curricula. This can help equip future nurses with the necessary skills and understanding to effectively implement and advocate for AAIs in pediatric care settings.
- The study highlights that owning a pet and prior exposure to AAIs positively influence nursing students' attitudes toward animals. Nursing programs should consider incorporating experiential learning opportunities that include direct interaction with animals, thereby fostering empathy and a deeper appreciation for the therapeutic potential of AAIs.
- Future research should focus on identifying specific educational gaps and the most effective methods for delivering AAI training to nursing students. By addressing these gaps, educators can better support nursing students in becoming competent and confident in using AAIs as a complementary approach to traditional pediatric care.

## 7 Conclusions

In conclusion, this study found that nursing students lacked sufficient knowledge about AAIs; however, they expressed belief in the effectiveness of such interventions and supported their use with children. These findings indicate a gap in formal education regarding AAIs, suggesting the need for structured training programs to enhance students' awareness, knowledge, and practical skills in this area. Integrating AAI‐related content into nursing curricula could help bridge this gap and better prepare future healthcare professionals for interdisciplinary approaches to patient care. Additionally, it was observed that owning a pet and prior exposure to AAI had a mediating effect on affection toward animals. This highlights the significant influence of personal experiences in shaping attitudes toward AAIs, suggesting that students with prior exposure may be more receptive to such interventions. Future studies could further explore how these experiences impact not only attitudes but also the willingness and confidence of nursing students to implement AAIs in clinical settings. Moreover, considering the growing recognition of AAIs in healthcare, further research could focus on identifying the most effective methods for integrating AAI education into nursing programs. Longitudinal studies examining the long‐term effects of AAI‐related education on clinical practice, patient outcomes, and interdisciplinary collaboration would provide valuable insights for curriculum development. By addressing these gaps, nursing education can better equip students with the necessary competencies to utilize AAIs effectively, ultimately improving patient care and well‐being.

## Author Contributions

**Abdullah Sarman:** conceptualization, writing – original draft, methodology, visualization, writing – review and editing, formal analysis, data curation. **Veysel Can:** conceptualization, formal analysis, methodology, visualization, writing – original draft. **Suat Tuncay:** conceptualization, data curation, formal analysis, methodology, writing – original draft, visualization. **Ahmed Loutfy:** conceptualization, formal analysis, methodology, visualization, writing – original draft.

## Ethics Statement

Ethics committee approval was secured from the Bingöl University Health Sciences Scientific Research and Publication Ethics Committee for the execution of the study (Approval No: 24.01.2024‐E.141855). Furthermore, requisite institutional permissions were obtained from the Dean's Office of the Faculty of Health Sciences for administering the questionnaires to student nurses (Approval No: 25.01.2024‐E.142284). Prior to commencing the study, student nurses were informed about its objectives, and their consent was duly obtained. The author(s) responsible for the Turkish validity, reliability, and adaptation study of the “Öney Animal Love Scale” were contacted via email, and permissions for the scale's usage were acquired. The entire process of this study adhered to the principles outlined in the Declaration of Helsinki.

## Consent

The students were informed about the purpose of the study. Informed consent has been approved by all participants.

## Conflicts of Interest

The authors declare no conflicts of interest.

## Data Availability

The data that support the findings of this study are available from the corresponding author upon reasonable request.
